# A high-speed, bright, red fluorescent voltage sensor to detect neural activity

**DOI:** 10.1038/s41598-019-52370-8

**Published:** 2019-11-04

**Authors:** Connor Beck, Yiyang Gong

**Affiliations:** Department of Biomedical Engineering, Duke University, Durham, NC 27708 USA

**Keywords:** Neuroscience, Optogenetics, Fluorescent proteins

## Abstract

Genetically encoded voltage indicators (GEVIs) have emerged as a technology to optically record neural activity with genetic specificity and millisecond-scale temporal resolution using fluorescence microscopy. GEVIs have demonstrated ultra-fast kinetics and high spike detection fidelity *in vivo*, but existing red-fluorescent voltage indicators fall short of the response and brightness achieved by green fluorescent protein-based sensors. Furthermore, red-fluorescent GEVIs suffer from incomplete spectral separation from green sensors and blue-light-activated optogenetic actuators. We have developed Ace-mScarlet, a red fluorescent GEVI that fuses Ace2N, a voltage-sensitive inhibitory rhodopsin, with mScarlet, a bright red fluorescent protein (FP). Through fluorescence resonance energy transfer (FRET), our sensor detects changes in membrane voltage with high sensitivity and brightness and has kinetics comparable to the fastest green fluorescent sensors. Ace-mScarlet’s red-shifted absorption and emission spectra facilitate virtually complete spectral separation when used in combination with green-fluorescent sensors or with blue-light-sensitive sensors and rhodopsins. This spectral separation enables both simultaneous imaging in two separate wavelength channels and high-fidelity voltage recordings during simultaneous optogenetic perturbation.

## Introduction

Genetically encoded voltage indicators (GEVIs) are a promising technology to probe neural circuitry and function. GEVIs enable recordings of neural activity with a combination of parallelism, relative non-invasiveness, temporal precision, and cell-type specificity^1–5^. If paired with the appropriate optics, existing GEVIs can simultaneously report voltage from dozens of neurons in a single field of view both *in vitro*^6^ and *in vivo*^7^. Recently developed GEVIs have millisecond timescale responses capable of producing high signal-to-noise ratio (SNR) readouts of single action potentials firing at frequencies as high as 100 Hz^8–11^. These tools are particularly advantageous for applications that quantify the number of spikes or the relative timing of spikes from individual neurons^7^. Furthermore, GEVIs enable cell-type specific experiments that investigate ensemble dynamics and spiking patterns from targeted, genetically defined subpopulations of cells^12^. However, existing GEVIs have the brightness and sensitivity to image only moderate numbers of neurons in a single field of view with cellular resolution. Therefore, it has been a longstanding goal of the neuroscience community to advance the signal-to-noise ratio of GEVIs in order to facilitate imaging an increased number of neurons with enhanced voltage sensitivity.

Recent generations of GEVIs have iteratively improved in key metrics such as sensitivity, brightness, and kinetics. One broad family of GEVIs is the series of sensors that fused a green fluorescent protein (GFP) to a variety of voltage sensing domains (VSDs) to produce bright emission that relayed the action of the VSD. These sensors have reported action potentials from neurons in culture, in slice, and *in vivo*. Existing FP configurations include: Voltage Sensitive Fluorescent Proteins (VSFPs)^13^ – a series of sensors that fused a voltage-sensitive phosphatase domain from *Ciona intestinalis* (CiVSP) to a fluorescent protein, including the first single-fluorescent protein GEVI, VSFP3.1^14,15^, Arclight^16^ – a sensor that fused a pH-sensitive mutant of GFP to CiVSP; Accelerated Sensor of Action Potentials (ASAP)^9,17^ – a sensor that inserted a circularly permuted super-folder GFP into a voltage-sensitive phosphatase domain to improve kinetics and sensitivity, and FRET-opsins – a recent class of sensors that takes advantage of FRET between a bright fluorescent protein and voltage-sensitive rhodopsin to produce sub-millisecond sensor kinetics^10^. Overall, the state-of-the-art green-fluorescent GEVIs can detect spikes with temporal precision that approaches the theoretical limit set by photon shot noise in multiple *in vivo* systems^18,19^.

While GEVIs based on green-fluorescent proteins and various voltage-sensitive domains have demonstrated fast kinetics and high spike detection fidelity *in vivo*, one element of the GEVI toolset that requires additional development is the class of red-fluorescent GEVIs. Expanding the spectral diversity of GEVIs would carry three benefits for the neuroscience community. First, a red-shifted fluorescent GEVI capable of resolving single action potentials would employ longer excitation and emission wavelengths than existing green-fluorescent GEVIs; longer wavelength operation would support lower phototoxicity and suffer less scattering, allowing deeper imaging in intact tissue. Such benefits were previously observed when using red-fluorescent calcium indicators^20,21^. Second, a red-fluorescent GEVI, in combination with existing green-fluorescent GEVIs, would enable investigations of the connections and interactions between distinct neural populations when using simultaneous, parallel imaging of two-color channels. Third, a red-fluorescent GEVI, in combination with existing blue-light-sensitive channelrhodopsins, would enable studies that probe the physiological effects of optical neural circuit manipulations. Photocurrent crosstalk in simultaneous imaging and optogenetic perturbation experiments is defined as the direct excitation of the rhodopsin via the voltage sensor illumination or from photocurrent driven by the voltage sensor itself. Simultaneous, spectrally separable optogenetic control and voltage imaging minimizes photocurrent crosstalk and thus prevents inadvertent influences on endogenous spiking activity.

Multiple red-fluorescent GEVIs exist, but they often lag behind their green-fluorescent counterparts in at least one key metric. The class of Archaerhodopsin (Arch)-based sensors is one promising class of red-fluorescent sensors. Because these Arch-based sensors have far-red emission, they are spectrally well separated from existing green-fluorescent sensors or light-activated ion channels. The first iteration of Arch sensors^22^ demonstrated large changes in fluorescence in response to changes in membrane potential, but had slow kinetics, which limited their ability to record fast trains of action potentials. Further engineering of this class of sensors improved fluorescence and kinetics but required high excitation intensity (800–8000 mW/mm^2^) to image^23–26^. Such high imaging intensities thus present challenges when scaling the optical recording to multiple neurons^6,26^. An alternate class of red-fluorescent GEVIs employed bright fluorescent proteins as the sensor readouts. For example, FlicR1^8^ fused CiVSP to a circularly permuted mApple. This sensor demonstrated relatively high voltage sensitivity in culture and mouse brain slice. More recently, a high-throughput screening process developed VARNAM^11^, a fusion of the Ace-D81S rhodopsin voltage sensing domain and mRuby3. These red-fluorescent GEVIs produced high SNR recordings at low to moderate excitation powers (15–100 mW/mm^2^). However, each of these red GEVIs experiences crosstalk photocurrent between the imaging and excitation light sources when used in conjunction with blue-light-activated rhodopsins. This photocurrent crosstalk arises from either direct excitation of the GEVI’s fluorophore by blue light (FlicR1) or weak activation of the rhodopsin by the yellow-green imaging illumination (VARNAM).

In this work, we developed Ace-mScarlet, a red-fluorescent GEVI that enables photocurrent-free, high SNR voltage imaging at red wavelengths. Like in the development of early red GECIs, we took advantage of an established sensing domain and optimized its performance with a red fluorescent protein^20,27^. Ace-mScarlet operates under the FRET-opsin mechanism employed by several existing GEVIs^10,11,28^. This configuration produces a fast, nearly linear change in fluorescence in response to changes in membrane potential; the sensor reported action potentials *in vitro* with high brightness and sensitivity. When paired with appropriate optical design and existing protein sensors or actuators, this new sensor enabled spectrally separated two-channel experiments such as multispectral imaging and simultaneous optogenetic “reading-and-writing” free of crosstalk photocurrent.

## Results

### Ace-mScarlet, a FRET-based red GEVI, exhibits high voltage sensitivity in HEK293T cells

**Figure 1:**
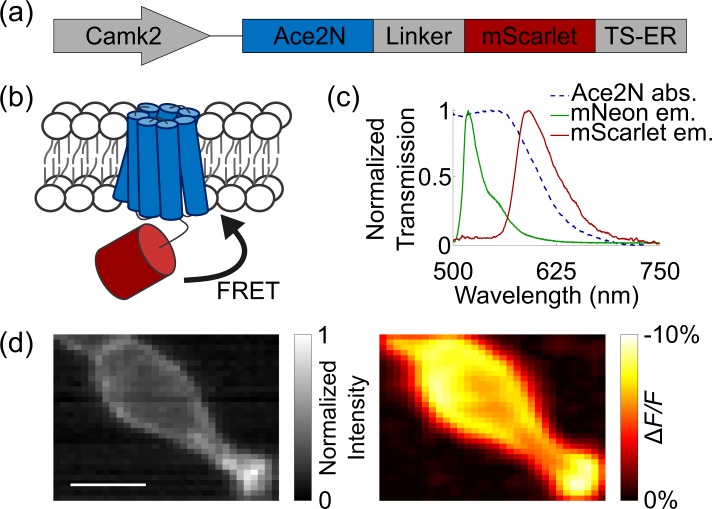
Ace-mScarlet senses membrane depolarization with increases in FRET and decreases in mScarlet emission. (**a)** Ace-mScarlet includes Golgi trafficking and endoplasmic reticulum export (TS-ER) sequences that improve membrane-localized expression. **(b)** Ace2N quenches a proportion of mScarlet’s emitted photons via FRET. **(c)** The absorption spectrum of Ace2N and emission spectra of mNeon and mScarlet have high overlap, potentially leading to high FRET efficiency. **(d)** *Left*: Fluorescence from a HEK293T cell expressing Ace-mScarlet; illumination intensity = 10 mW/mm^2^; *Right*: Δ*F/F* response of the same cell to a +100 mV depolarizing voltage step. Scale bar: 10 μm.

Our construct’s design was inspired by the FRET-opsin configuration used in the development of Ace2N-mNeon^10^, which took advantage of fluorescence resonance energy transfer (FRET) between the fast, voltage-sensitive rhodopsin Ace and bright, green fluorescent protein mNeonGreen. This FRET configuration enables voltage-sensitive changes in emission intensity. Ace2N-mNeon owes its high spike detection fidelity and speed to Ace’s high voltage sensitivity and sub-millisecond kinetics. We adopt the same sensor architecture by using Ace2N for our new construct and fused it to the bright, red fluorescent protein mScarlet (Fig. 1a). In addition to high voltage sensitivity, Ace2N drives virtually no steady-state photocurrent, consistent with previously developed GEVIs that employ Ace mutants^10,11^ (Supp. Fig. 1). Like Ace2N-mNeon, Ace-mScarlet’s voltage sensitivity depends on the FRET efficiency between the voltage-sensitive acceptor and fluorescent protein donor; an increase in a neuron’s membrane potential increases Ace’s absorbance, which results in higher quenching of mScarlet’s emission (Fig. 1b,d).

The voltage sensitivity of FRET-opsin sensors depends critically on the baseline FRET efficiency. Previous fluorescence-lifetime microscopy measurements experimentally found that Ace2N-mNeon’s FRET efficiency is 25%^29^. Using this figure and mScarlet’s photophysical properties, we predicted that Ace-mScarlet’s FRET efficiency is slightly lower at 19% (*Methods*). This slight decrease in FRET efficiency results from multiple competing factors. Ace’s peak absorption is better aligned with mNeonGreen than with mScarlet (Fig. 1c), suggesting that Ace-mScarlet’s FRET efficiency would be lower than Ace2N-mNeon’s FRET efficiency. This effect is balanced both by Ace’s broad absorption spectrum and by the spectral overlap integral’s stronger weighting of longer wavelengths. In addition, mNeonGreen has a higher quantum yield (0.80) compared to mScarlet (0.70)^30,31^. Nevertheless, the two fluorescent proteins have comparable quantum yield, and mScarlet has the highest quantum yield among all monomeric red-fluorescent proteins^31^.

**Figure 2:**
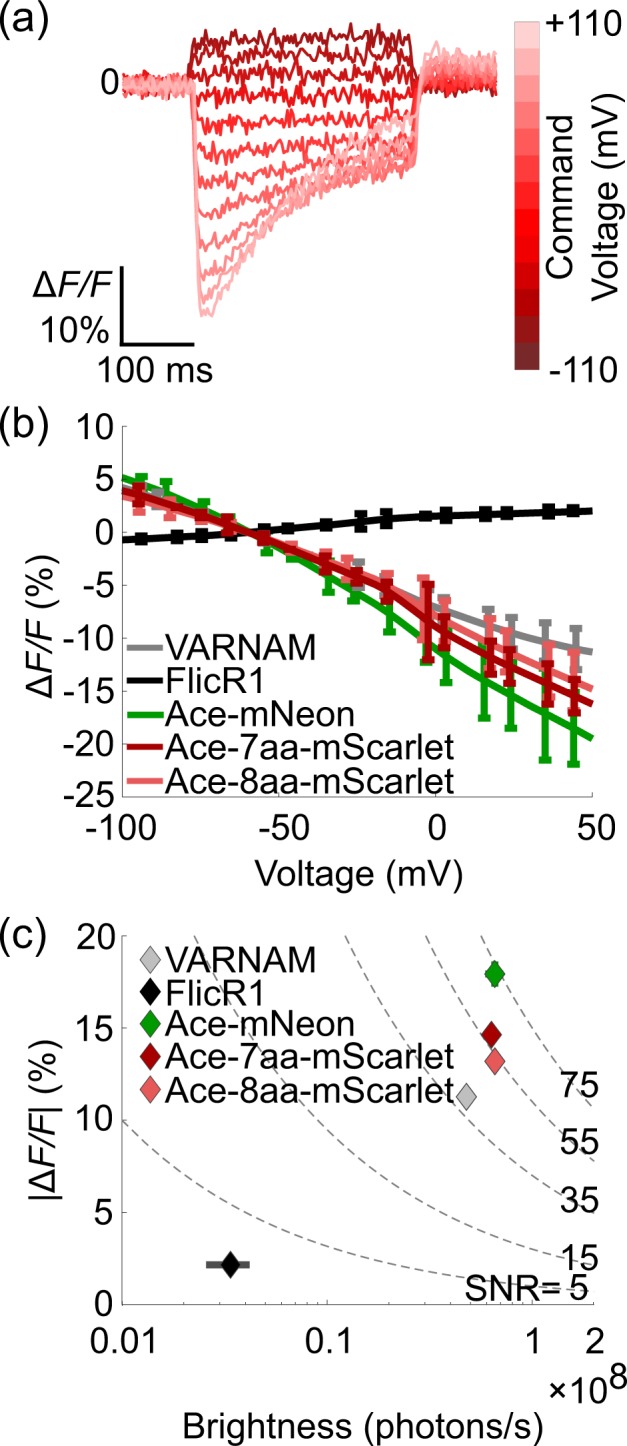
HEK293T cells expressing Ace-mScarlet exhibit high voltage sensitivity and brightness under whole-cell patch clamp. (**a)** Optical response of HEK293T cells transfected with Ace-mScarlet in response to a series of step voltages. We held HEK293T cells at –65 mV at the start of each sweep and applied command voltages ranging from −70 mV to +150 mV relative to the baseline voltage. **(b)** The peak fluorescence response of Ace-mScarlet decreases nearly linearly as a function of membrane voltage (mean ± s.d.). **(c)** The peak fluorescence response to a +100 mV step (from −70 mV to +30 mV) vs. brightness for HEK cells expressing Ace-7aa-mScarlet and Ace-8aa-mScarlet (*n* = 52 cells), Ace2N-mNeon (*n* = 23 cells), VARNAM (*n* = 42 cells), and FlicR1 (*n* = 30 cells). Ace-7aa-mScarlet and Ace-8aa-mScarlet exhibited the highest SNR of all red GEVIs tested (points and error bars are mean ± s.e.m.). Dashed lines represent SNR isocontours (*Methods*).

We next engineered the baseline FRET efficiency by modulating the distance between the Ace2N acceptor and fluorescent protein donor; FRET efficiency, and thus voltage sensitivity, increases as the distance between domains decreases. Manipulating this separation introduces a trade-off between voltage sensitivity and baseline brightness: short linkers facilitate high FRET, and therefore increase sensitivity, while long linkers sacrifice sensitivity for brightness. We sought to optimize this trade-off to maximize our sensor’s SNR, defined as $\Delta F/F\times \sqrt{{F}_{0}}$, where Δ*F*/*F* is the relative change in fluorescence produced by a defined change in voltage and *F*_0_ is the sensor’s baseline fluorescence. To maximize Ace-mScarlet’s SNR, we screened peptide linkers ranging from 1 to 18 amino acids to manipulate the distance between Ace and mScarlet (Table 1). We expressed each variant in Human Embryonic Kidney (HEK293T) cells under the CamKIIα promoter within a lentiviral backbone^10^ and tested their voltage sensitivities using whole-cell patch clamp under 565 nm illumination light at 10 mW/mm^2^. We applied a series of 20 mV voltage steps ranging from −70 mV to +150 mV relative to a –65 mV holding potential (Fig. 2a). These fluorescence responses to voltage steps revealed each variant’s Δ*F/F* response per 100 mV (Supp. Fig. 2, *Methods*). Ace-mScarlet exhibited a nearly linear relationship between membrane voltage and fluorescence for all variants (Fig. 2b). The trend between sensor brightness, voltage sensitivity, and linker length map out the expected trade-off: the shortest linker variant produced the most sensitive response: −14.9 ± 0.8% Δ*F/F* (mean ± s.e.m.; *n* = 23) per 100 mV. The longest-linker variant produced the brightest baseline fluorescence with moderately less sensitivity.

**Table 1:** Linker composition of Ace-mScarlet variants.

Variant	Peptide linker amino acids
Ace-1aa-mScarlet	L
Ace-2aa-mScarlet	LR
Ace-3aa-mScarlet	MLR
Ace-4aa-mScarlet	KMLR
Ace-5aa-mScarlet	MLRSL
Ace-6aa-mScarlet	KMLRSL
Ace-7aa-mScarlet	KMLRSGL
Ace-8aa-mScarlet	KMLRGSGL
Ace-9aa-mScarlet	RARDPPVAT
Ace-18aa-mScarlet	MKASSRRTISQNKRRHVV

We focused our characterization on two specific Ace-mScarlet variants – the seven amino acid linker (KMLRSGL) and eight amino acid linker (KMLRGSGL) variants were simultaneously bright and sensitive. The combination of high voltage sensitivity and brightness from the seven and eight amino acid linker variants together yielded superior SNR compared to other variants: 57.3 ± 2.5 (mean ± s.e.m.; *n* = 52) and 52.7 ± 2.4 (mean ± s.e.m.; *n* = 52), respectively (*p* < 0.05 for all variants, two-sided Wilcoxon rank test; Fig. 2c, Supp. Fig. 2). Due to their high SNR relative to other Ace-mScarlet variants and other red-fluorescent GEVIs, we used the 7 amino acid linker variant, Ace-7aa-mScarlet, and the 8 amino acid linker variant, Ace-8aa-mScarlet, for all subsequent experiments. These two variants had comparable voltage sensitivity to each other: in response to a +100 mV voltage step, Ace-7aa-mScarlet produced a Δ*F/F* of –14.6 ± 0.3% (mean ± s.e.m.; *n* = 52) and Ace-8aa-mScarlet produced a Δ*F/F* of –13.2 ± 0.3% (mean ± s.e.m.; *n* = 52). However, each variant slightly underperformed Ace2N-mNeon’s –17.9 ± 0.7% response (mean ± s.e.m.; *n* = 23). The two constructs had higher sensitivity than other red-fluorescent GEVIs FlicR1 (Ace-7aa-mScarlet: *p* = 2 × 10^−10^; Ace-8aa-mScarlet: *p* = 2 × 10^−10^; one-sided Wilcoxon rank test) and VARNAM (Ace-7aa-mScarlet: *p* = 1 × 10^−12^; Ace-8aa-mScarlet: *p* = 8 × 10^−6^; one-sided Wilcoxon rank test). The two constructs were also nearly as bright as Ace2N-mNeon (Ace-7aa-mScarlet: *p* = 0.39; Ace-8aa-mScarlet: *p* = 0.57; one-sided Wilcoxon rank test; Fig. 2c) and had similar on- and off-kinetics (Ace-mScarlet: *n* = 12 for each variant; Ace2N-mNeon: *n* = 16; *p* > 0.15 for all comparisons, two-sided Wilcoxon rank test; Table 2).

**Table 2:** Ace-mScarlet has comparable on- and off-kinetics to Ace2N-mNeon.

	On-kinetics			Off-kinetics		
	${\tau }_{{\rm{fast}}}$ (ms)	${\tau }_{{\rm{slow}}}$ (ms)	${P}_{{\rm{fast}}}$ (%)	${\tau }_{{\rm{fast}}}$ (ms)	${\tau }_{{\rm{slow}}}$ (ms)	${P}_{{\rm{fast}}}$ (%)
Ace2N-mNeon	0.69 ± 0.12	3.9 ± 1.7	69.8 ± 3.8	1.5 ± 0.33	6.5 ± 1.9	64.8 ± 4.4
Ace-7aa-mScarlet	0.79 ± 0.18	2.4 ± 0.6	79.4 ± 1.0	1.1 ± 0.32	8.6 ± 2.8	58.0 ± 4.4
Ace-8aa-mScarlet	0.80 ± 0.21	4.0 ± 2.0	69.0 ± 8.3	1.0 ± 0.29	6.9 ± 2.8	52.9 ± 7.5

### Ace-mScarlet’s responds to single action potentials with high SNR changes in fluorescence in cultured hippocampal rat neurons

**Figure 3:**
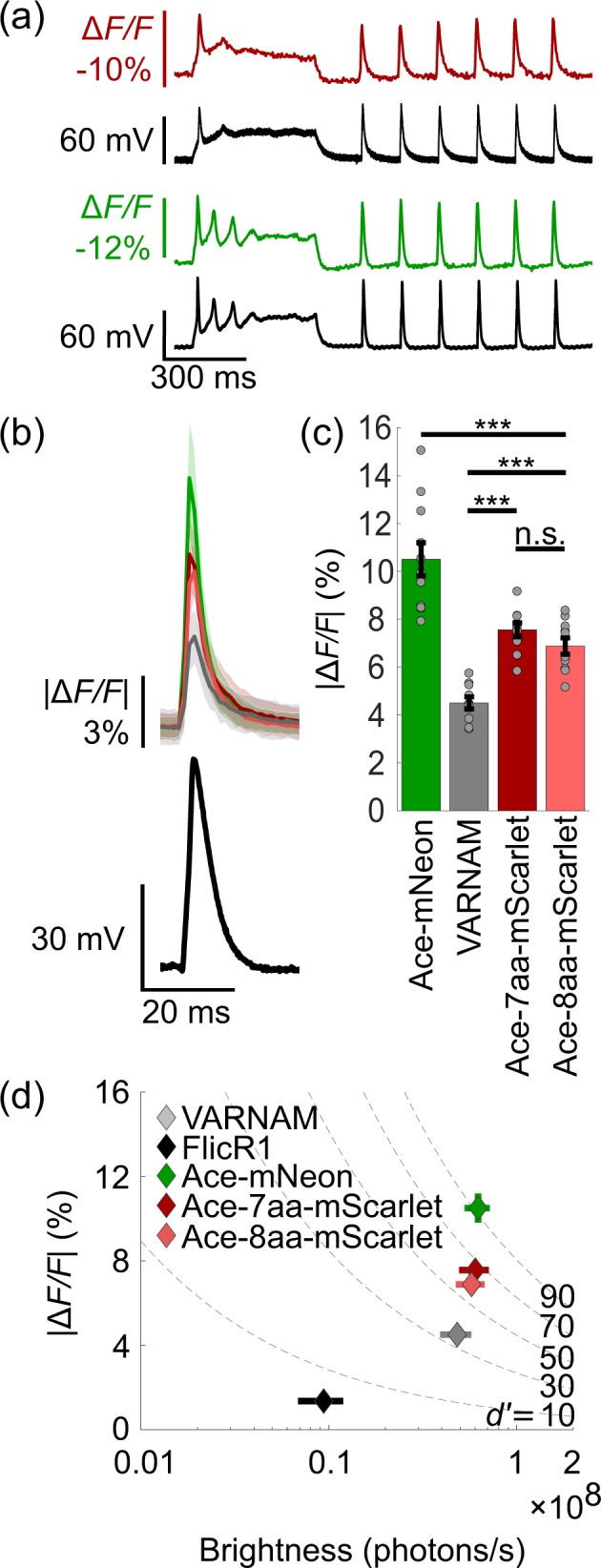
Ace-mScarlet reports single action potentials in cultured neurons with high sensitivity and brightness. **(a)** Representative fluorescence traces from Ace-mScarlet (*top*) and Ace2N-mNeon (*bottom*) faithfully track single action potentials and subthreshold changes in potential in simultaneous electrophysiology recordings. **(b)** Each sensor’s average fluorescence traces (*colored solid lines*) in response to single action potentials (*n* = 180 spikes from 6 neurons each; shaded region represents mean ± s.d.) closely match the average waveform of electrically recorded action potentials (*black line*). **(c)** Ace-7aa-mScarlet’s, Ace-8aa-mScarlet’s, VARNAM’s, and Ace2N-mNeon’s average peak response to action potentials (*n* = 10 neurons each; mean ± s.e.m.). **(d)** Δ*F/F* vs. brightness of Ace-7aa-mScarlet, Ace-8aa-mScarlet, Ace2N-mNeon, VARNAM (*n* = 10 neurons each; mean ± s.e.m.) and FlicR1 (*n* = 5 neurons; mean ± s.e.m.) in response to single action potentials. Ace-mScarlet variants had both higher sensitivity and brightness than other red GEVIs. Dashed lines represent *d’* isocontours (*Methods*).

We next characterized the Ace-mScarlet variants with the highest SNR in cultured hippocampal rat neurons to evaluate their response to single action potentials. Ace-7aa-mScarlet and Ace-8aa-mScarlet tracked individual action potentials with high temporal resolution and kinetics comparable to Ace2N-mNeon (Fig. 3a,b). Ace-7aa-mScarlet and Ace-8aa-mScarlet produced –7.6 ± 0.3% and –6.9 ± 0.3% Δ*F/F*, respectively (mean ± s.e.m.; *n* = 10). In comparison, VARNAM produced –4.5 ± 0.3% Δ*F/F* (mean ± s.e.m.; *n* = 10), FlicR1 produced 1.4 ± 0.04% Δ*F/F* (mean ± s.e.m.; *n* = 5), and Ace2N-mNeon produced –10.5 ± 0.7% Δ*F/F* (mean ± s.e.m.; *n* = 10), in response to single action potentials (Fig. 3c). Ace-mScarlet was nearly as bright as Ace2N-mNeon in neurons (Ace-7aa-mScarlet: *p* = 0.35; Ace-8aa-mScarlet: *p* = 0.25, one-sided Wilcoxon rank test).

To comprehensively characterize each sensor’s ability to report action potentials, we used an established metric for spike discriminability, *d*’, which simultaneously captures a sensor’s sensitivity, brightness, and kinetics when responding to spikes at the shot-noise limit^10,11,18^. Ace-7aa-mScarlet and Ace-8aa-mScarlet produced *d’* values of 79 ± 12 (mean ± s.e.m.; *n* = 10) and 76 ± 14 (mean ± s.e.m.; *n* = 10), respectively, a testament to their high sensitivity and brightness (Fig. 3d). Consequently, the increase in Ace-mScarlet’s *d’* corresponded to a predicted combined false positive and negative spike detection rate of 2.9 × 10^−47^ errors per frame for Ace-7aa-mScarlet and 1.4 × 10^−39^ errors per frame for Ace-8aa-mScarlet. In traditional metrics of performance, Ace-mScarlet enabled action potential detection with SNR = 29 ± 3 (mean ± s.e.m.; *n* = 10) using the 7aa linker variant, and SNR = 26 ± 3 (mean ± s.e.m.; *n* = 10) using the 8aa linker variant, which were approximately 2× higher than that of VARNAM and 14× higher than that of FlicR1, respectively.

### Ace-mScarlet enables multispectral voltage imaging with simultaneous calcium imaging or optogenetic stimulation

We next demonstrated the utility of Ace-mScarlet for parallel, spectrally multiplexed experiments in combination with green-fluorescent sensors or blue-light-activated channelrhodopsins. These multi-channel voltage imaging applications were not possible when using green-fluorescent GEVIs, whose excitation spectra have high overlap with existing sensors or rhodopsins. This overlap would introduce high fluorescence crosstalk between the excitation and imaging components of dual-channel experiments. This fluorescence crosstalk in dual-channel imaging experiments is defined as the spectral overlap of the fluorescence signal of each sensor in each imaging channel. In simultaneous optogenetic perturbation and imaging experiments, fluorescence crosstalk is defined as the direct excitation of mScarlet by the rhodopsin’s blue excitation light. Ace-mScarlet, however, reports changes in membrane potential with high SNR with red-shifted excitation and emission spectra. This red shift has the potential to minimize both fluorescence crosstalk with green fluorescent sensors and photocurrent crosstalk with blue-light-excited optogenetic actuators.

**Figure 4:**
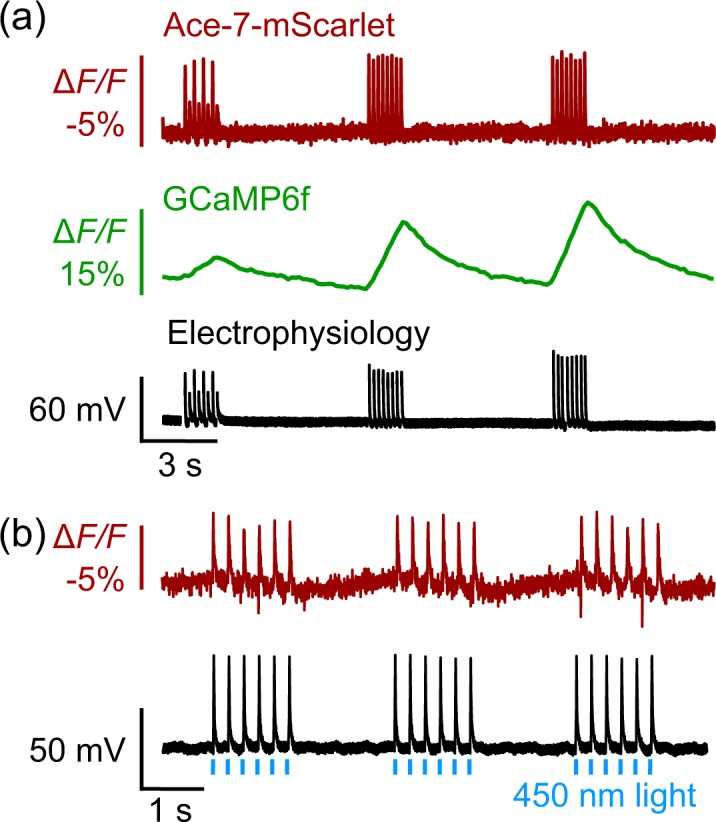
Ace-mScarlet enables dual channel experiments using either green calcium indicators or blue-light-activated rhodopsins with minimal crosstalk. **(a)** Ace-7aa-mScarlet (*top*) and GCaMP6f (*middle*) facilitate parallel voltage and calcium recording in cultured neurons with minimal fluorescence crosstalk. Ace-mScarlet’s kinetics closely track individual action potentials (*bottom*) at 8 Hz. **(b)** Ace-mScarlet produces spikes within corrected fluorescent traces (*top*) that closely match action potentials elicited via optogenetic stimulation of CheRiff (*bottom*) with 8 Hz, 2 ms pulses of blue light.

We sought to demonstrate Ace-mScarlet’s spectral separation from green-fluorescent indicators for simultaneous calcium and voltage imaging. We co-transfected dissociated rat hippocampal neurons with the green calcium indicator GCaMP6f^32^ and Ace-7aa-mScarlet. Using whole-cell current clamp, we applied multiple rounds of near-threshold, 2 ms electrical stimuli at 8 Hz. These stimuli produced electrical action potentials, which in turn produced distinguishable fluorescence transients within GCaMP6f and Ace-mScarlet recordings in the green and red channels, respectively (Fig. 4a). While GCaMP6f produced a temporally broad calcium transient in the green imaging channel in response to each burst of spikes, Ace-mScarlet’s fast kinetics distinguished the individual spikes elicited by each of the 8 electrical stimulation pulses in the red imaging channel. Furthermore, our voltage indicator reported waveforms corresponding to subthreshold voltage fluctuations, while the same transients escaped detection by calcium indicators (Fig. 4a).

Another useful capability of high SNR, red-fluorescent GEVIs is the ability to record changes in membrane voltage elicited by simultaneous optical stimulation via optogenetic actuators such as channelrhodopsins with virtually no photocurrent crosstalk and minimal fluorescence crosstalk between the rhodopsin’s excitation LED and Ace-mScarlet imaging channel. We demonstrated this capability with our Ace-mScarlet sensor and the blue-light-sensitive channelrhodopsin CheRiff^26^. We co-transfected rat hippocampal neuron cultures with Ace-7aa-mScarlet and CheRiff-EGFP. Rather than imaging Ace-mScarlet at 565 nm as described above, we shifted the imaging wavelength to 585 nm. This shift in excitation wavelength eliminated incidental excitation of the rhodopsin by the imaging LED while maintaining high SNR. Upon blue light excitation, CheRiff drove robust photocurrent, whereas the onset of 585 nm light did not elicit photocurrent in CheRiff-expressing cells (Supp. Fig. 3). We drove action potentials in CheRiff-positive neurons using 1 ms pulses of 450 nm light at 1.5 mW/mm^2^. Ace-mScarlet produced −4.8 ± 0.1% Δ*F/F* in response to CheRiff-driven action potentials (mean ± s.e.m.; *n* = 12 cells, 30 spikes per cell; Fig. 4b). Ace-mScarlet’s voltage sensitivity was independent of expression levels between neurons co-expressing CheRiff compared to neurons expressing Ace-mScarlet alone (Supp. Fig. 4). We observed transient, positive changes in Ace-mScarlet fluorescence coincident with blue light excitation that directly excited mScarlet’s fluorophore (Supp. Fig. 5a,b). These transients were typically between 1 and 15% Δ*F/F* (Supp. Fig. 6a–c), but these effects were less pronounced at shorter pulse durations and shorter excitation wavelengths (Supp. Fig. 6c). Additionally, mScarlet underwent moderate photoswitching upon blue light excitation which caused a slight increase in baseline fluorescence (Supp. Fig. 6b). To effectively remove these direct excitation artifacts, we applied a blind algorithm to correct fluorescence traces (Supp. Fig. 5a–d, *Methods*). In short, this method quantified the transient fluorescent artifact for each optical stimulation waveform in HEK293T cells. This method then removed artifacts from the optical recordings of action potentials from neurons by interpolating the average template and subtracting the average expected artifact from the recording. This method generalized to both 1 ms and 10 ms optical stimulation waveforms (Supp. Fig. 5e, *Methods*); it virtually eliminated transient artifacts on average and reduced noise evoked by blue light excitation of mScarlet.

## Discussion

Ace-mScarlet enables a new class of multispectral and high-speed optogenetic experiments on large scales. We can now perform voltage imaging and optogenetic stimulation in parallel at moderate excitation powers of 7–10 mW/mm^2^ with high SNR and cellular resolution without unintentionally perturbing the targeted neural populations’ intrinsic membrane potential dynamics via photocurrent driven by blue-light-sensitive rhodopsins upon voltage sensor excitation at steady-state (Supp. Figs 1 and 3). Upon green-light excitation, Ace-mScarlet drove small but quantifiable transient photocurrent (Supp. Fig. 1). However, like other FP-based GEVIs that utilize the Ace sensing domain, our sensor produced no steady-state photocurrent and thus enables continuous recordings of voltage.

Our advancement will enable a continued trajectory toward simultaneous imaging and stimulation of ever larger populations of neurons in culture. Such imaging on larger scales will facilitate precise interrogation of neural circuits consisting of multiple neural populations. Our ability to perform fully spectrally separable two-channel experiments at moderate excitation power stems from two properties within Ace-mScarlet’s design: (1) the position of mScarlet’s red-shifted absorption spectrum relative to the blue-shifted excitation spectrum of the rhodopsin, which minimizes photocurrent crosstalk between the imaging LED and rhodopsin; and (2) complementary optical design, which further capitalizes on the separation between CheRiff’s excitation spectrum and Ace-mScarlet’s absorption spectrum.

First, Ace-mScarlet enables a new regime of photocurrent-free voltage sensing due to its high spectral separation from blue-light-activated rhodopsins. The pairing of Ace-mScarlet with CheRiff supports high intensity excitation of the sensor that achieved low shot-noise measurements of voltage while entirely avoiding the activation of blue-light-sensitive rhodopsin ion channels (Supp. Fig. 3). Many previous studies have employed several strategies and protein tools to achieve spectral separation between the actuator and sensor. For example, the commonly employed pairing of a green sensor with a red-light-activated rhodopsin enables high SNR imaging at green wavelengths with minimal photocurrent crosstalk from green or yellow excitation^33–36^. However, this paradigm employs high intensity blue light excitation that could inadvertently activate the broad, blue-shifted tail within the excitation spectra of many red-light-sensitive opsins even when using wavelengths as short as 490 nm^37,38^.

Second, our results highlight the importance of optical design in conjunction with engineered protein tools. Previous studies employed a red-fluorescent sensor and blue-light-sensitive rhodopsin, but each of these studies had unique trade-offs in performance vs. crosstalk photocurrent. Arch-based sensors had substantial spectral separation between the GEVI and channelrhodopsin, which eliminated imaging-induced photocurrent, but required substantially higher imaging excitation powers than FP-based voltage sensors^25,26^. Existing red-fluorescent FP-based GEVIs imaged voltage with high SNR at lower excitation powers than rhodopsin-only GEVIs, but still reported tens of picoampere level crosstalk between the stimulation and imaging channels^8,11^. This remaining photocurrent crosstalk occurred in part due to the 561 nm imaging laser that partially overlapped with the activation spectra of blue-shifted rhodopsins. In this work, we achieved photocurrent-free imaging by taking advantage of Ace-mScarlet’s broad excitation spectrum and high brightness with tailored imaging design: we imaged our sensor at a wavelength red-shifted and away from mScarlet’s peak excitation wavelength. This red-shifted excitation scheme retained high-SNR voltage recordings and simultaneously eliminated incidental excitation of the rhodopsin by the imaging illumination (Supp. Fig. 3). Ace-mScarlet improves on the state-of-the-art by achieving high SNR recordings of voltage with more flexibility – the high fidelity of Ace-mScarlet enables lower, but still relatively high, SNR voltage imaging at sub-optimal excitation wavelengths away from the peak excitation wavelength of mScarlet. Our scheme is an example of joint optical and protein deployment where spectral design takes advantage of the protein sensor’s broad excitation spectrum and high brightness. In the future, the continued improvement of GEVIs’ photophysical properties will thus also engender concomitant improvements in optical design that take advantage of these properties.

Although the pairing of a blue-light-sensitive ion channel and red sensor greatly reduced the possibility for photocurrent crosstalk from the sensor excitation to the stimulation channel, the pair increased the possibility for the optical stimulation excitation to introduce fluorescence crosstalk artifacts into the imaging channel. We observed these low amplitude, transient increases in the fluorescence of the red imaging channel during blue light stimulation of our rhodopsin. These transients are due to mScarlet’s non-negligible absorption at blue wavelengths. While these transients had consistent temporal profiles, their amplitude varied depending on excitation power, duration, excitation wavelength, and mScarlet’s expression level from cell to cell. To address this fluorescence crosstalk caused by direct excitation of mScarlet by blue light, we developed a generalizable, blind algorithm that automatically predicts the occurrence of fluorescence artifacts. We characterized fluorescence transients elicited by pulses of 450 nm light. We then applied our strategy to remove artifacts originating from these defined optogenetic manipulations (*Methods*). Because our method successfully removed artifacts originating from both low intensity, long duration or high intensity, pulsed stimuli, our method is likely generalizable when correcting for artifacts originating from arbitrary stimulation patterns generated by the experimentalist.

Our configuration of Ace-mScarlet accesses one specific regime within the trade-off between sensitivity and brightness associated with all FRET-opsin sensors but maximized SNR within this trade-off. Overall, the maximum SNR of the Ace-mScarlet variants was lower than that of the Ace2N-mNeon variants. This decrease in SNR is most likely due to Ace and mScarlet having lower FRET efficiency than Ace and mNeon. Such a photophysical behavior is expected for two reasons: Ace’s absorption spectrum better overlaps with mNeon than with mScarlet, and mNeon’s quantum yield is higher than that of mScarlet. Nevertheless, we surprisingly observed statistically comparable brightness between Ace2N-mNeon and Ace-mScarlet. We rationalize this observation as a result of the two sensors’ different linkers between the rhodopsin and fluorescent protein. Ace-mScarlet employs a longer peptide linker (7–8 amino acids) between the FRET acceptor and donor domains than Ace2N-mNeon (2 amino acids). The longer optimal linker length of Ace-mScarlet thus produced a sensor that favored brightness as opposed to FRET efficiency or voltage sensitivity. Overall, the optimal linker lengths of the Ace-mScarlet and Ace2N-mNeon variants suggest that different steric considerations arise from using various fluorescent protein donors in the FRET-opsin configuration. These considerations result in optimal sensors in different regimes of the brightness vs. sensitivity trade-off.

Ace-mScarlet expands the growing family of red GEVIs and more accurately detects spikes than previously developed red GEVIs. Although its relative response to single action potentials was only a modest increase relative to state-of-the-art red GEVIs, Ace-mScarlet’s enhanced brightness facilitated significantly higher SNR. This high SNR resulted in a linear increase in spike detection fidelity (*d’*)^18^. Such linear increases in *d*’ significantly reduce the errors associated with detecting false spikes or failing to detect true spikes by orders of magnitude – the false positives and false negative rates decrease super-exponentially with *d’*. For example, Ace-mScarlet’s two-fold increase in SNR over VARNAM translates to over 30 orders of magnitude lower spike detection errors per hour. This super-exponential reduction in spike detection errors promotes voltage imaging’s utility for long term imaging experiments with virtually no false positives or negatives.

Our work explores the non-intuitive interaction between a voltage-sensitive rhodopsin’s proton pumping capabilities and the corresponding FRET-opsins’ voltage sensitivity. The critical mutations governing both the voltage sensitivity and function of the Ace rhodopsin proton pump are the manipulations of the D81 residue. These mutations not only modulate the rhodopsin’s steady-state photocurrent, but influence its voltage sensitivity in FRET-opsin configurations in a way unique to each rhodopsin-FP pairing: VARNAM (Ace-D81S-mRuby3) performs better than Ace-D81N-mRuby3, but our Ace-mScarlet, which uses the D81N substitution previously used in Ace2N-mNeon, performs better than Ace-D81S-mScarlet with the same peptide linkers (Supp. Fig. 2). These results contradict the prevailing theory that one variant of the Ace domain should be more voltage sensitive than another variant independent of the fused fluorescent protein. Our results motivate further studies of pairings between mutations of this residue and FRET donors within the FRET-opsin configuration. Specifically, future studies could more deeply explore the relationship between mutations at this critical residue and the FRET efficiency between these Ace mutants and mScarlet or mRuby3. A difference in FRET efficiency between mutant Ace domains and red FPs could explain the unexpected differences in performance we observed and produce sensors with higher sensitivity, brightness, or kinetics.

## Methods

### Plasmid construction

We constructed all Ace-mScarlet plasmids using overlap PCR to introduce specific peptide linkers between Ace2N and mScarlet. All constructs except FlicR1 were expressed in a lentivirus backbone under the CamkIIα promoter. A second overlap assembly was used to fuse the TS-ER sequence to the 3′ end of all Ace-mScarlet variants. FlicR1 was expressed under the CAG promoter in a pcDNA3.1 backbone to increase expression to detectable levels at lower excitation power. Ace-7aa-mScarlet and Ace-8aa-mScarlet are deposited on Addgene as plasmids #129701 and #129702, respectively.

### Cell cultures

HEK293T cells were cultured in Dulbecco Modified Eagle Medium (DMEM) supplemented with 10% fetal bovine serum (FBS) and 1% streptomycin. We transfected HEK293T cells with Lipofectamine 1–2 days after passaging and imaged them 2 days post-transfection.

We dissected rat hippocampal neurons from postnatal day 0 Sprague-Dawley pups (Charles River Labs) and cultured them in Neurobasal Media A supplemented with GlutaMAX and B-27 Supplement. We transfected neurons using calcium phosphate 3 days after plating. We then imaged and patched these neurons 3–5 days post-transfection.

### Electrophysiology

We applied various current and voltage waveforms to whole-cell patched HEK293T cells and cultured rat hippocampal neurons using an Axon Digidata 1550A (Axon Instruments) digitizer, Multiclamp 700A amplifier (Axon Instruments), and pClamp software. In HEK cells, we applied voltage steps in 20 mV increments from −70 mV to +150 mV relative to a −65 mV holding potential. In neurons, we applied 300 ms pulses of current steps ranging from 10–100 pA followed by suprathreshold, 2 ms pulses of current ranging from 300 to 1500 pA at 8 Hz for 700 ms. Stimulus artifacts in simultaneous voltage and calcium imaging electrophysiology traces have been manually removed.

All samples were mounted in a perfusion chamber in which the extracellular media was kept at 22 °C and consisted of 150 mM NaCl, 4 mM KCl, 10 mM glucose, 10 mM HEPES, 2 mM CaCl_2_, and 2 mM MgCl_2_. The intracellular solution contained 129 mM K-gluconate, 10 mM KCl, 10 mM HEPES, and 4 mM Na_2_ATP. We applied a *post hoc* correction for the junction potential.

### Optics

We used a 40 × 0.8 NA water immersion objective (Nikon) for all imaging. We imaged cells at 400 Hz using an sCMOS camera (QImaging, OptiMOS). We imaged Ace-mScarlet and VARNAM with a 565 nm LED (Thorlabs, M565L3) with an intensity of 10 mW/mm^2^ at the sample plane. We imaged FlicR1 using a 532 nm laser (Thorlabs, CPS532) with an intensity of 100 mW/mm^2^ at the sample plane. To image each red-fluorescent sensor, we used a Nikon G-2E/C filter cube containing a 540/24 nm excitation filter, 565 nm longpass dichroic mirror, and 620/60 nm emission filter. To image Ace2N-mNeon, we used a 490 nm LED (Thorlabs, M490L4) with an intensity of 10 mW/mm^2^ at the sample plane and a Nikon B-2E/C filter cube containing a 480/30 nm excitation filter, 505 nm longpass dichroic mirror, and a 535/40 nm emission filter.

For simultaneous dual-channel imaging experiments, we used the same 565 nm LED and 490 nm LED as above to image Ace-mScarlet and GCaMP6f, respectively. These LEDs passed through a 585/11 nm excitation filter (Semrock, FF01-585/11-25) and 475/28 nm excitation filter (Semrock, FF01-475/28-25), respectively. The blue light intensity was 10 mW/mm^2^ at the sample plane, while the yellow light intensity was 7 mW/mm^2^ at the sample plane. Emitted fluorescence passed through a multiband dichroic mirror (Semrock, FF409/493/596-Di02-25×36) before entering a Photometrics DV2 channel splitter, which separated green and red emission via a 565 nm longpass dichroic mirror (Chroma, T565lpxr). The green fluorescence passed through a 525/45 nm emission filter (Semrock, FF01-525/45-25), while the red fluorescence passed through a 641/75 nm emission filter (Semrock, FF01-641/75-25).

For simultaneous optogenetic stimulation of CheRiff and voltage imaging with Ace-mScarlet, we applied 2 ms pulses of 450 nm LED (Thorlabs, M450LP1) light at 8 Hz for 700 ms with an intensity of 1.5 mW/mm^2^ at the sample. The blue excitation light passed through a 436/20 nm excitation filter (Chroma, ET436/20×) and reflected off a 511 nm long-pass dichroic mirror (Semrock, FF511-Di01-25×36) to join with the Ace-mScarlet excitation path. We imaged Ace-mScarlet using the same LED, light intensity, and excitation/emission filters as in the dual-channel imaging experiments.

### Δ*F/F* and SNR calculation

We defined Δ*F/F* as the difference between the fluorescence during an applied voltage step and at rest. For all recordings, we defined the shot-noise limited SNR as $(\Delta F/F)\times \sqrt{{F}_{0}}$. This definition of SNR also generated the isocontouors for Fig. 2. To calculate the SNR for each cell, we generated a mask using the top 30% of pixels by measured by SNR on a pixel-wise basis. These pixels then generated the fluorescence traces for all Ace-mScarlet, Ace2N-mNeon, VARNAM, and FlicR1 cells; these traces then produced the sensor's $\varDelta F/F$ and ${F}_{0}$. For GCaMP6f data, we manually drew masks over the cell to produce the fluorescence traces.

To determine the theoretical false positive rate of our sensor, we used an established parameter from signal detection theory *d’*^18^. This metric considers not only Δ*F/F*, but the shape of the action potential waveform and shot noise of the imaging system to produce a more comprehensive measure of spike detection fidelity than SNR alone. We used an approximation of the metric ${d}^{{\rm{^{\prime} }}}\approx {\rm{S}}{\rm{N}}{\rm{R}}\times \sqrt{(\tau /2)}$, which assumes an exponentially decaying sensor response with time constant τ, to generate the isocontours for Fig. 3.

### FRET efficiency estimate

Based on Ace2N-mNeon’s measured FRET efficiency using fluorescence-lifetime imaging microscopy^29^, we estimated Ace-mScarlet’s FRET efficiency based on the following FRET equations:

$$E=\frac{1}{1+{(\frac{r}{{R}_{0}})}^{6}}$$

where *r* represents the distance between the FRET acceptor and FRET donor and ${R}_{0}^{6}$ is:

$${R}_{0}^{6}=\frac{9\,\mathrm{ln}(10)}{128{\pi }^{5}{N}_{A}}\frac{{\kappa }^{2}{Q}_{D}}{{n}^{4}}J$$

where ${\kappa }^{2}$ represents the dipole orientation factor between the FRET donor and acceptor, ${Q}_{D}$ is the quantum yield of the FRET donor, *n* is the index of refraction of the medium, and

$$J={\int }^{}\overline{{f}_{D}}(\lambda ){{\epsilon }}_{A}(\lambda ){\lambda }^{4}d\lambda $$

represents the overlap between the normalized FRET donor emission spectrum ($\overline{{f}_{D}}(\lambda )$) and the FRET acceptor absorption spectrum (${{\epsilon }}_{A}$). We based our estimate of Ace-mScarlet’s FRET efficiency on the differences between mNeonGreen’s and mScarlet’s quantum yields, each FP’s emission spectrum’s overlap with Ace’s absorption spectrum, and the assumption that the dipole orientation factor ${\kappa }^{2}$ and distance between the FRET domains remains constant.

### Transient artifact subtraction from mScarlet blue-light excitation

We recorded the fluorescent artifact in HEK293T cells expressing mScarlet in the same dual channel stimulation and imaging configuration described above; we used the same LEDs, filters, recording rates, and 1 ms or 10 ms pulses of blue light. We then identified the camera frames (recorded at 400 Hz) with fluorescent artifacts and matched them to the onset of the blue stimulation light (recorded at 10 kHz) driven by the patch-clamp protocol. We averaged these individual artifacts to generate an artifact template waveform elicited by either the 1 ms and 10 ms pulses of blue light (Supp. Fig. 5a). We then corrected fluorescence traces from dual channel stimulation and imaging experiments in neurons using the template waveform in four steps. First, we again used the fast, electronic activation signal of the LEDs as the temporal reference to accurately locate the onset of the fluorescence transient. Second, we used the temporal offset between the onset of the blue LED and the subsequent camera frame to interpolate this high acquisition rate template waveform (acquired at 10 kHz) to the fluorescence waveform (acquired at 400 Hz) (Supp. Fig. 5b). Third, we matched the interpolated template to the maximum value of the fluorescence trace within the onset of the blue LED (Supp. Fig. 5c). Finally, we subtracted the template waveform from the selected frames (Supp. Fig. 5c,d).

### Ethical approval

The Duke Institutional Animal Care and Use Committee (IACUC) approved all animal experiments. All experiments were conducted in accordance with the guidelines and regulations within such an approval.

## Supplementary information

Supplementary Information

## Data Availability

Data will be made available from the corresponding author upon reasonable request.
