# Supplementary material for: A high-speed, bright, red fluorescent voltage sensor to detect neural activity
Source: Sci Rep. 2019 Nov 4;9:15878. doi: 10.1038/s41598-019-52370-8 (PMC6828731; doi:10.1038/s41598-019-52370-8)
Supplement: Supplementary file 1 — Supplementary Information [file 41598_2019_52370_MOESM1_ESM.pdf]

A high-speed, bright, red fluorescent voltage sensor to detect neural activity

Connor Beck, Yiyang Gong*

Dept. of Biomedical Engineering, Duke University, Durham, NC, 27708, USA

*Corresponding author: yiyang.gong@duke.edu

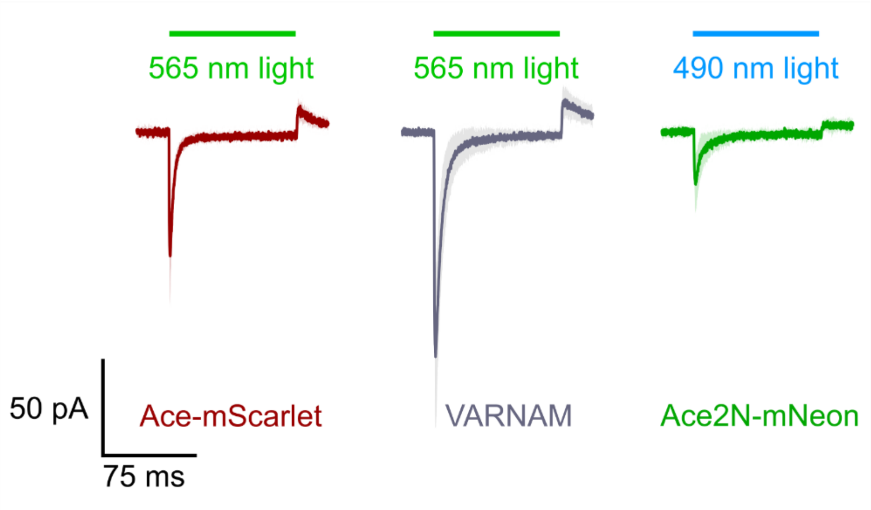

Supplemental Figure 1. Ace variants do not drive steady-state photocurrent after excitation by the imaging LED.

Average photocurrent (*solid lines*) elicited by 100 ms pulses of 565 nm light in HEK293T cells expressing Ace-7aa-mScarlet (*left*; n = 12 cells, 120 pulses of light) and VARNAM (*middle*; n = 12 cells, 120 pulses of light) or by 490 nm light in HEK293T cells expressing Ace2N-mNeon (*right*; n = 8 cells, 80 pulses of light). The average steady-state photocurrent is –1.8 ± 0.9 pA for Ace-mScarlet (n = 12 cells; mean ± s.d.), –1.9 ± 2.5 pA for VARNAM (n = 12 cells; mean ± s.d.), and –1.0 ± 1.1 pA for Ace2N-mNeon (n = 16 cells; mean ± s.d.) and $p > 0.05$ for all pairwise comparisons of these sensors' steady-state photocurrent (two-sided Wilcoxon rank test). Shaded regions represent mean ± s.d.

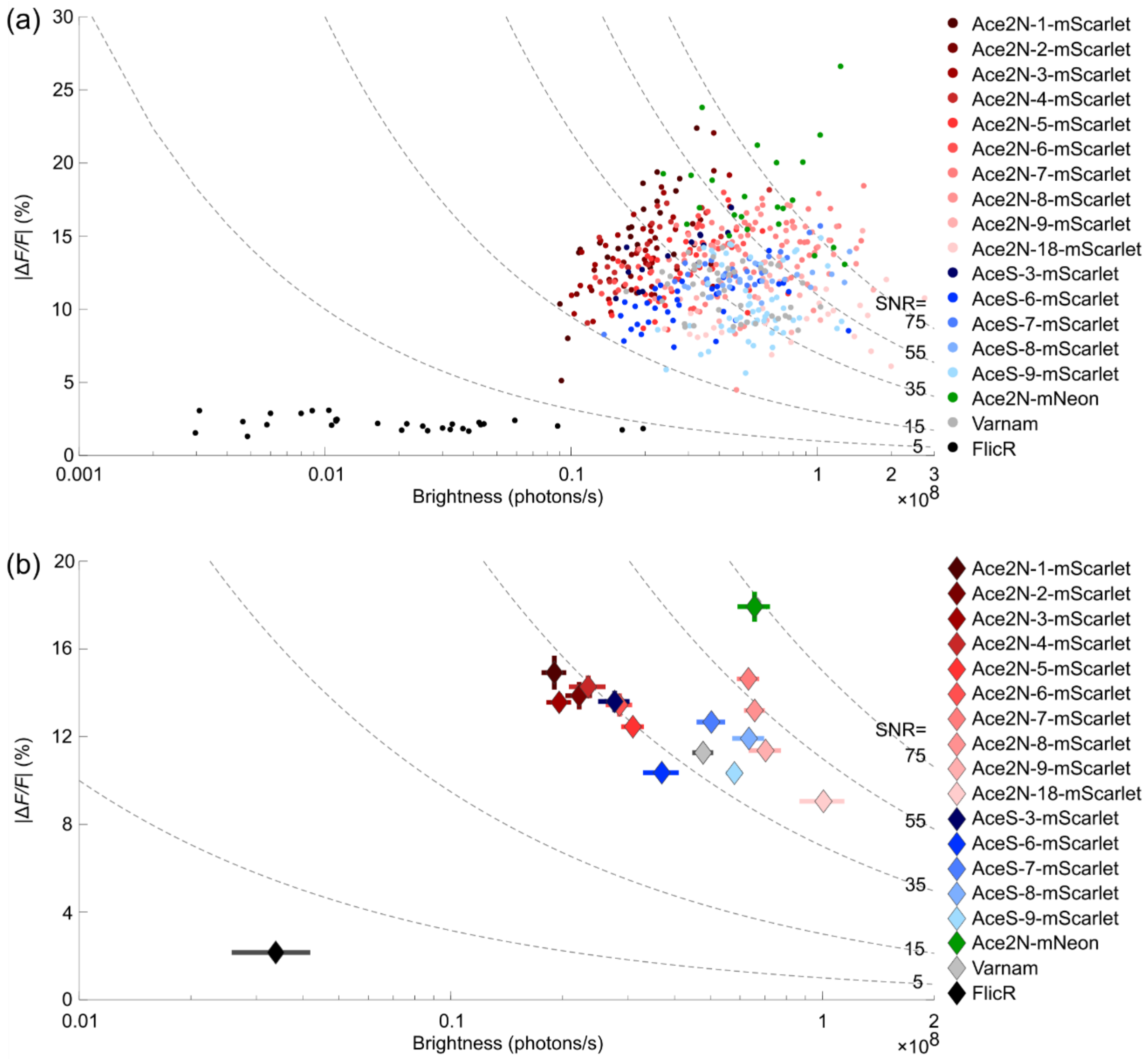

Supplemental Figure 2. Ace-mScarlet variants with different peptide linkers trade off brightness for sensitivity as the linker length increases.

(a) The peak fluorescence response to a +100 mV step (from –70 mV to +30 mV) vs. brightness for individual HEK293T cells expressing Ace2N-mScarlet variants with linkers ranging from 1 to 18 amino acids, (1aa, *n* = 23; 2aa, *n* = 22; 3aa, *n* = 33; 4aa, *n* = 21; 5aa, *n* = 39; 6aa, *n* = 22; 7aa, *n* = 52; 8aa, *n* = 52; 9aa, *n* = 24; 18aa, *n* = 23 cells), Ace-D81S-mScarlet variants with linkers ranging from 3 to 9 amino acids (3aa, *n* = 14; 6aa, *n* = 35; 7aa, *n* = 23; 8aa, *n* = 24; 9aa, *n* = 54), Ace2N-mNeon (*n* = 23), VARNAM (*n* = 42), and FlicR1 (*n* = 30). Ace-7aa-mScarlet and Ace-8aa-mScarlet exhibited the highest SNR of all red GEVIs tested. Dashed lines represent SNR

isocontours (*Methods*). **(b)** Average fluorescence and brightness for each variant in **(a)**. Points and error bars are mean ± s.e.m.

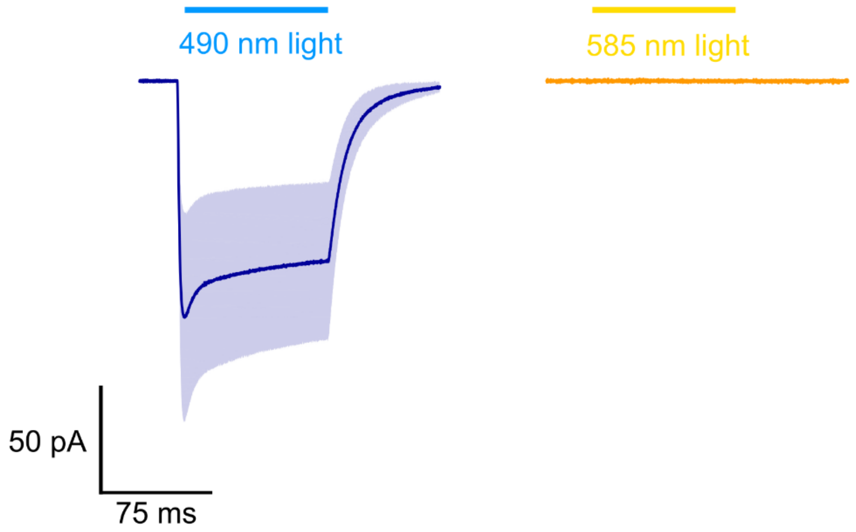

Supplemental Figure 3. Ace-mScarlet enables crosstalk-free excitation of CheRiff. Average photocurrent (*solid lines*) elicited by 100 ms pulses of 490 nm light (*left*; n = 8 cells, 80 pulses of light) and 585 nm light (*right*; n = 5 cells, 50 pulses of light) in HEK293T cells expressing CheRiff-EGFP. Steady-state current during 585 nm excitation does not differ from the holding current (p = 0.48, two-tailed Wilcoxon rank test). Shaded regions represent mean ± s.d.

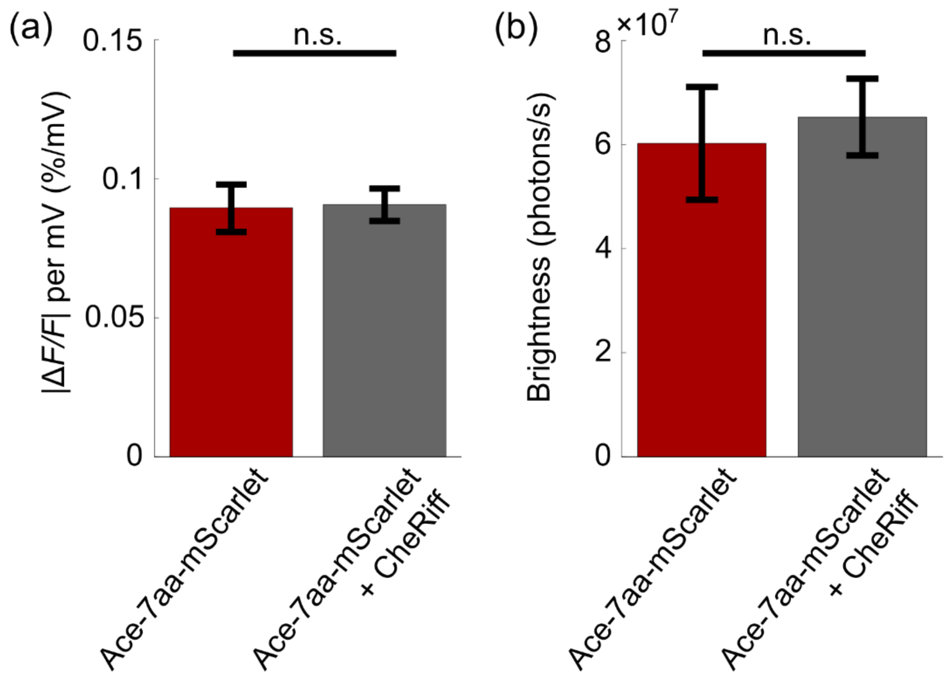

Supplemental Figure 4: Ace-mScarlet's expression and voltage sensitivity are not significantly affected by co-expression with CheRiff. (a) Ace-7aa-mScarlet's Δ*F/F* per mV during an action potential in cultured neurons is not significantly different when Ace-7aa-mScarlet is expressed by itself or when it is expressed simultaneously with CheRiff (n = 8 cells, p = 0.16; two-sided Wilcoxon rank test). **(b)** The mean brightness of Ace-7aa-mScarlet in cultured neurons is not significantly different when Ace-7aa-mScarlet is expressed by itself or when it is expressed simultaneously with CheRiff (n = 8 cells, p = 0.21; two-sided Wilcoxon rank test). Error bars represent mean ± s.e.m.

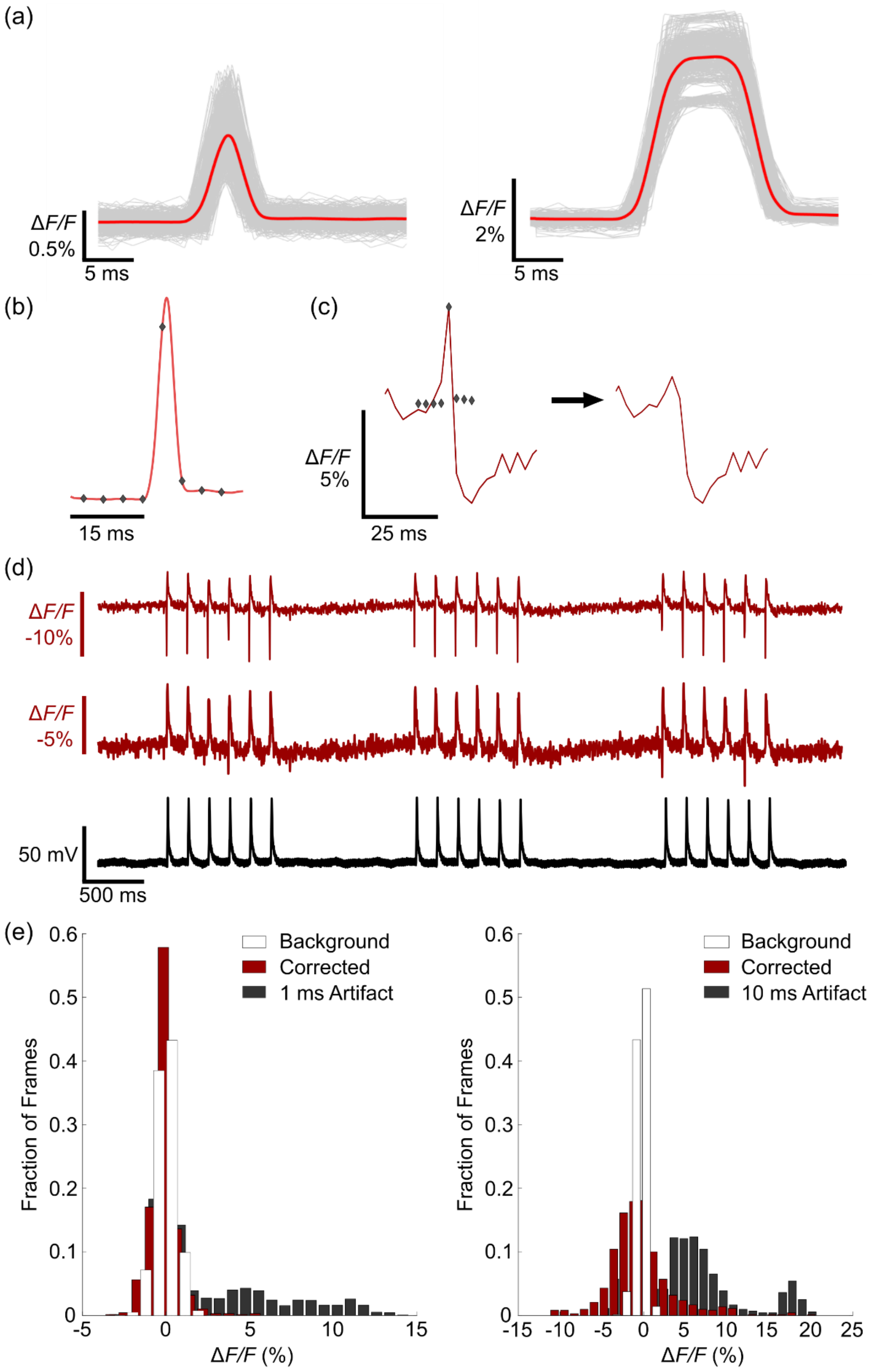
(a)
ΔF/F
0.5%
5 ms
ΔF/F
2%
5 ms
(b)
15 ms
(c)
ΔF/F
5%
25 ms
(d)
ΔF/F
-10%
ΔF/F
-5%
50 mV
500 ms
(e)
Background
Corrected
1 ms Artifact
Fraction of Frames
ΔF/F (%)
Background
Corrected
10 ms Artifact
Fraction of Frames
ΔF/F (%)

Supplemental Figure 5. Our blind algorithm identifies and removes fluorescent artifacts caused by direct 450 nm excitation of mScarlet to improve spike detection.

(a) Individual (*gray*) and average (*red*) fluorescent artifacts caused by direct excitation of mScarlet by 1 ms pulses of 450 nm light (*left*; n = 15 cells, 450 pulses of light) and 10 ms pulses of 450 nm light (*right*; n = 15 cells, 450 pulses of light). The full width at half maximum (FWHM) of the average artifact is 3.0 ms for 1 ms pulses of light and is 12.7 ms for 10 ms pulses. **(b)** To apply the average artifact identified in (a), we interpolated the template waveform to the corresponding frames in the fluorescence trace based on the offset between the blue LED onset and camera frames. The gray diamonds superimposed on the template waveform are a representative interpolation matched to one specific fluorescence artifact. **(c)** We scaled the interpolated template to the maximum value of the artifact in the fluorescence trace, aligned this scaled template to the onset of the blue LED (*left*) and subtracted this scaled, interpolated template from the trace (*right*). **(d)** Fluorescence trace of a spiking neuron co-expressing Ace-7aa-mScarlet and CheRiff before (*top*) and after (*middle*) artifact subtraction. The electrophysiology is shown in black (*bottom*). **(e)** Distributions of the $\Delta F/F$ noise from the frames that encompass the blue-light-elicited transient artifact before and after correction (n = 15 cells, 450 spikes) for 1 ms pulses (*left*) and 10 ms pulses (*right*) of 450 nm light. For 1 ms pulses, our artifact subtraction algorithm removes the stimulation artifact by improving the frames' $\Delta F/F$ distribution during 1 ms blue light excitation from $\Delta F/F$ = 2.3 ± 3.7% without correction to –0.1 ± 0.8% with correction (mean ± s.d.). The baseline noise for these recordings was –0.02 ± 0.6% (mean ± s.d.). For 10 ms pulses of 450 nm light, our algorithm improves the frames' $\Delta F/F$ distribution during blue light excitation from $\Delta F/F$ = 5.9 ± 5.8% to –0.4 ± 3.8% (n = 6 cells, 180 spikes; mean ± s.d.). The baseline noise for these recordings was –0.3 ± 0.6% (mean ± s.d.).

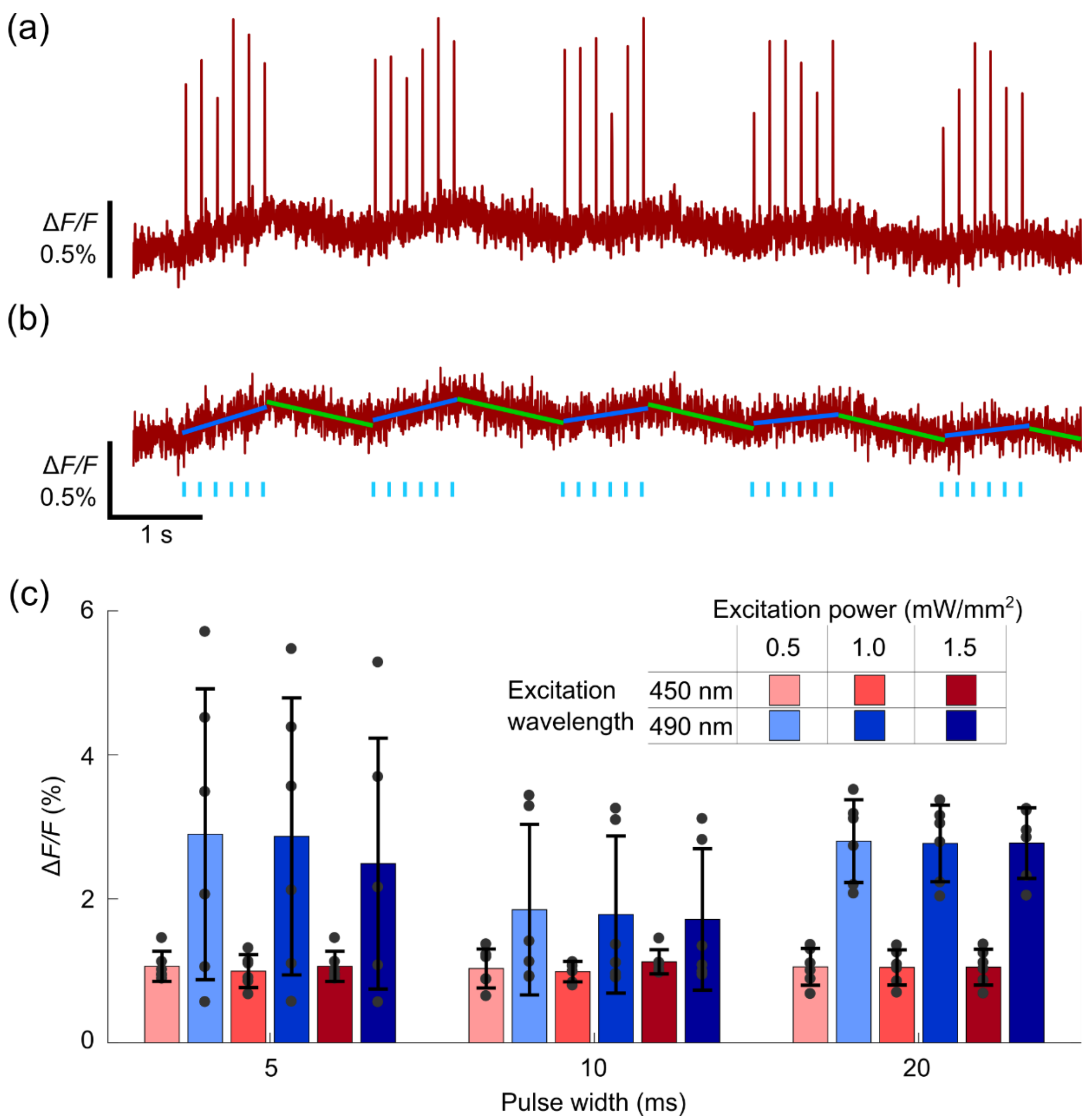

Supplementary Figure 6. mScarlet undergoes direct excitation and mild photoswitching upon blue light illumination.

(a) Sample fluorescence trace of a HEK293T cell expressing CMV-mScarlet upon excitation with 1 ms pulses of 450 nm light at 1.5 mW/mm^2 at the sample delivered at 8 Hz. Direct excitation of mScarlet causes transient, millisecond-scale, positive changes in fluorescence. **(b)** The same trace as in (a), but with the transient fluorescence removed. mScarlet undergoes mild photoswitching during periods with the pulsed blue light excitation, which causes a positive trend in its baseline fluorescence. Blue lines represent linear fits to the baseline fluorescence during the 800 ms periods of pulsed blue light excitation. The average slope of the baseline fluorescence

during blue light excitation was 0.09 ± 0.09%/s (*n* = 15 cells, mean ± s.d.). Green lines represent linear fits to the baseline fluorescence during the 1100 ms periods without blue light excitation. The average mScarlet photobleaching rate in the absence of blue light excitation was –0.10 ± 0.06%/s (*n* = 15 cells, mean ± s.d.). **(c)** Average height of the fluorescent artifacts elicited in HEK293T cells expressing CMV-mScarlet upon varying excitation conditions (*n* = 6 cells, 30 pulses of light each). Shorter pulses of light at shorter excitation wavelengths elicited lower amplitude artifacts than longer, higher powered stimulus conditions ($p < 10^{-4}$ for all comparisons between pulses elicited by 450 nm and 490 nm light; one-sided Wilcoxon rank test).
